# Perioperative sleep apnea: a real problem or did we invent a new disease?

**DOI:** 10.12688/f1000research.7218.1

**Published:** 2016-01-11

**Authors:** Sebastian Zaremba, James E. Mojica, Matthias Eikermann

**Affiliations:** 1 Department of Anaesthesia Critical Care and Pain Medicine, Massachusetts General Hospital and Harvard Medical School, Boston, Massachusetts, 02114, USA; 2 Department of Neurology, Rheinische-Friedrich-Wilhelms-University, Bonn, D-53127, Germany; 3 German Center for Neurodegenerative Diseases, Bonn, D-53127, Germany; 4 Department of Pulmonary and Critical Care Medicine, Massachusetts General Hospital and Harvard Medical School, Boston, Massachusetts, 02114, USA; 5 Department of Anaesthesia and Critical Care, University Hospital Essen, Essen, 45147, Germany

**Keywords:** Obstructive sleep apnea, Perioperative sleep apnea, Bariatric surgery, Upper airway, Anaesthesiology

## Abstract

Depending on the subpopulation, obstructive sleep apnea (OSA) can affect more than 75% of surgical patients. An increasing body of evidence supports the association between OSA  and perioperative complications, but some data indicate important perioperative outcomes do not differ between patients with and without OSA. In this review we will provide an overview of the pathophysiology of sleep apnea and the risk factors for perioperative complications related to sleep apnea. We also discuss a clinical algorithm for the identification and management of OSA patients facing surgery.

## Objectives

Perioperative sleep apnea is becoming a major concern for anesthesiologists and intensivists ^1^. However, similar to another problem in the perioperative field (i.e. residual paralysis) ^2^, the negative impact of sleep apnea on postsurgical management and patient outcomes remains unclear. In this article, we will (1) summarize currently available data on the prevalence and pathophysiology of sleep apnea in the perioperative context, (2) discuss the bidirectional effect of anesthesia and surgery on sleep apnea, and (3) suggest a clinical pathway for the perioperative identification and management of sleep apnea patients that is being used by many physicians at the Massachusetts General Hospital in Boston, MA, USA based on in-house discussions and input we received from Dr Shiroh Isono, Chiba, Japan during his visits in our institution.

## Obstructive sleep apnea and why it is important in the perioperative setting

### Definition and Epidemiology

Obstructive sleep apnea (OSA) is characterized by recurrent episodes of reduction or cessation of airflow despite continued or increased respiratory effort. Hypopneas are shallow breaths resulting from partial obstruction and reduced intraluminal diameter of the upper airway (UA). Apneas are characterized by the absence of airflow due to complete airway collapse. These respiratory events are associated with oxyhemoglobin desaturations, neuronal arousal, disrupted sleep, and impaired daytime functioning ^3^.

Based on daytime symptoms, the incidence of OSA in the general population ranges from 0.3% to 5% ^4– 6^. However, several studies investigating the prevalence of OSA without daytime symptoms using polysomnography (PSG) found much higher rates ^7– 9^ ( Table 1A) with undiagnosed OSA in up to 80% of patients ^10^. Furthermore, with obesity representing a major risk factor for OSA, one can expect a higher prevalence of sleep apnea as rates of obesity continue to climb ^11– 13^.

**Table 1.** Important publications on the incidence of sleep apnea in the general population (A) and in different groups of surgical patients (B).

Reference	Population studied	Age [yrs.]	Gender	Sample size	Method	Definition of OSA	OSA rate
*A) General population*							
Young, T. *et al.* 1993 ^9^	State employees in Wisconsin	30–60	both	602	PSG	AHI > 5/h	9% w, 24% m
Bixler, E.O. *et al.* 2001 ^8^	telephone households in southern Pennsylvania	≥ 20	women	1000	PSG	AHI ≥ 15/h	2.2%
Durán, J. *et al.* 2001 ^7^	residents of Vitoria-Gasteiz, Spain	30–70	both	2148	Interview/PSG	AHI ≥ 5/h	14.5% w, 27.0% m
Vozoris, N.T. 2012 ^6^	2005–06 and 2007–08 NHANES cohort		both	12,593	questionnaire	Diagnosis of OSA	4.3%
*B) Surgical populations*							
Fidan, H. *et al.* 2006 ^164^	All surgical patients		both	433	PSG	AHI > 5/h	3.2%
Finkel, K.J. *et al.* 2009 ^165^	All surgical patients	43–68	both	2877	questionnaire/sleep study (n=217)	AHI > 5/h	5.9% (23.7%)
* Ramachandran, S.K. *et al.* 2010 ^139^	All surgical patients	> 18	both	3884	Chart review	Diagnosis of OSA	7.2%
* D'Apuzzo, M.R. *et al.* 2010 ^166^	Orthopedic surgery patients		both	258,455	Chart review	Diagnosis of OSA	6.4%
Weingarten, T.N. *et al.* 2010 ^14^	Bariatric surgery patients	> 18	both	797	PSG	AHI > 5/h	77.5%
* Mokhlesi, B. *et al.* 2013 ^34^	Bariatric surgery patients	44.2 ± 11.8	both	91,028	Chart review	Diagnosis of OSA	36.0%
* Griffin, J.W. *et al.* 2013 ^16^	Orthopedic surgery patients	68.8 ± 11.6	both	22,988	Chart review	Diagnosis of OSA	5.9%
Amra, B. *et al.* 2014 ^167^	CABG surgery patients	58.6 ± 11.1	both	61	BERLIN questionnaire	High OSA risk	40.9%
Uchôa, C.H. *et al.* 2015 ^168^	CABG surgery patients	57.4 ± 7.5	both	67	PSG	AHI > 15/h	56.0%
* Wong, J.C. *et al.* 2015 ^169^	CABG surgery patients		both	545	Chart review	Diagnosis of OSA	13.0%
Foldvary-Schaefer, N. *et al.* 2015 ^55^	Cardiac surgery patients		both	107	PSG	AHI > 5/h	73.8%

* = retrospective analysis; OSA = obstructive sleep apnea; AHI = apnea hypopnea index; w = women; m = men; NHANES = National Health and Nutrition Examination Surveys; PSG = polysomnography; CABG = coronary artery bypass graft

In the perioperative population, the prevalence of OSA varies widely among different subgroups ( Table 1B). Bariatric surgery patients, the subpopulation most extensively studied, have been shown to have rates of up to 77.5% ^14^. Many of these patients are asymptomatic despite severe sleep apnea ^15^. Other surgical populations, such as orthopedic surgery patients, have been shown to have rates of OSA that are only slightly higher than the general population ^16^. This broad range of prevalence rates may be related to the diverse distribution of risk factors for OSA, such as obesity based on a high body mass index (BMI), age, and/or comorbidities (e.g. stroke and myocardial infarction). Further research is needed to evaluate if the surgical population has a higher risk of OSA independent of these factors.

### Perioperative consequences of sleep apnea

In the general population, OSA is a risk factor for diabetes ^17^ and cardiovascular diseases including cardiac arrhythmias ^18, 19^, myocardial infarction ^20^, pulmonary hypertension ^21^, systemic hypertension ^19, 22^, heart failure ^19^, renal disease ^23^, stroke, and death ^24^. Proposed mechanisms include hypoxemia, sympathetic activation, metabolic dysregulation, left atrial enlargement, endothelial dysfunction, systemic inflammation, and hypercoagulability ^25^.

Perioperative complications occur more often in patients with OSA compared to controls ^26– 29^. These include delirium ^30, 31^, reintubation, pneumonia ^32– 35^, atrial arrhythmias, myocardial infarction, and pulmonary embolism ^36^ ( Table 2). Delirium in the postoperative period is associated with increased morbidity and mortality ^37, 38^, as well as long-term cognitive and functional decline ^39, 40^. These complications increase utilization of intensive care and length of stay as described in a recent retrospective study in 1,058,710 patients undergoing elective surgeries ^35^. Our group also found that, independent of OSA, reintubation and unplanned intensive care unit (ICU) admission may result in a substantial increase in in-hospital mortality ^41, 42^.

**Table 2.** Effect of obstructive sleep apnea on the risk for postoperative complication after non-upper-airway surgery.

Consequence	Reference	Sample	N Total (OSA pos.)	Effect size OR (95% CI)
1) Reintubation	Memtsoudis, S. *et al.* 2011 ^32^	Orthopedic surgery patients	234,152 (58,538)	OR 1.95 (1.91 to 1.98)
		General surgery patients	182,186 (45,545)	OR 5.20 (5.05 to 5.37)
	Mokhlesi, B. *et al.* 2013 ^34^	Bariatric surgery patients	91,028 (33,196)	OR 4.35 (3.97 to 4.77)
	Mokhlesi, B. *et al.* 2013 ^35^	Orthopedic surgery patients	783,723 (43,502)	OR 14.3 (13.3 to 15.3)
		Prostate surgery patients	67,848 (2779)	OR 10.3 (8.0 to 13.3)
		Abdominal surgery patients	79,101 (2633)	OR 2.01 (1.7 to 2.4)
		Cardiovascular surgery patients	128,038 (6006)	OR 1.80 (1.65–1.95)
	Abdelsattar *et al.* 2015 ^36^	Gen. and vascular surgery patients; *untreated vs. treated* *OSA*	2,646 (1181)	OR 2.5 (n. reported)
2) Unplanned ICU admission	Kaw *et al.* 2012 ^28^	Non-cardiac surgery patients	471 (189)	OR 4.4 (n. reported)
	Chia, P. *et al.* 2013 ^170^	Elective surgery patients	5,432 (338)	OR 2.2 (1.1 to 4.6)
3) Hypoxemia	Kaw *et al.* 2012 ^28^	Non-cardiac surgery patients	471 (189)	OR 7.9 (n. reported)
4) Pneumonia	Mokhlesi, B. *et al.* 2013 ^35^	Orthopedic surgery patients	783,723 (43,502)	OR 1.06 (0.94 to 0.19)
		Prostate surgery patients	67,848 (2779)	OR 1.3 (0.74 to 2.30)
		Abdominal surgery patients	79,101 (2633)	OR 0.71 (0.56 to 0.92)
		Cardiovascular surgery patients	128,038 (6006)	OR 0.85 (0.72 to 1.01)
	Memtsoudis, S. *et al.* 2011 ^32^	Orthopedic surgery patients	234,152 (58,538)	OR 1.37 (1.33 to 1.41)
		General surgery patients	182,186 (45,545)	OR 1.41 (1.35 to 1.47)
5) Delirium	Flink B. *et al.* 2012 ^31^	Orthopedic surgery patients; ≥ 65 yrs.	105 (15)	OR 4.3 (1.2 to 15.8)
	Roggenbach, J. *et al.* 2014 ^171^	Elective cardiac surgery patients	92 (9)	OR 6.8 (2.6 to 15–4) *for* *AHI ≥ 19/h*
8) Myocardial infarction	Abdelsattar *et al.* 2015 ^36^	General and vascular surgery patients; *untreated vs. treated* *OSA*	2646 (1181)	OR 2.6 (n. reported)
7) Atrial fibrillation	Mokhlesi, B. *et al.* 2013 ^34^	Bariatric surgery patients	91,028 (33,196)	OR 1.25 (1.11 to 1.41)
8) Pulmonary embolism	Memtsoudis, S. *et al.* 2011 ^32^	Orthopedic surgery patients	234,152 (58,538)	OR 0.90 (0.84 to 0.97)
		General surgery patients	182,186 (45,545)	OR 1.22 (1.15 to 1.29)
7) Longer LOS	Kaw *et al.* 2012 ^28^	Non-cardiac surgery patients	471 (189)	OR 1.7 (n. reported)
	Memtsoudis, S.G. *et al.* 2014 ^33^	Orthopedic surgery patients	530,089 (44,246)	OR 1.1 (1.1 to 1.2)
8) Mortality	Griffin, J.W. 2013 ^16^	Shoulder arthroplasty patients	22,996 (1983)	OR 1.083 (n. reported)
	Mokhlesi, B. *et al.* 2013 ^34^	Bariatric surgery patients	91,028 (33,196)	OR 0.34 (0.23 to 0.50)
	Mokhlesi, B. *et al.* 2013 ^35^	Orthopedic surgery patients	783,723 (43,502)	OR 0.65 (0.45 to 0.95)
		Prostate surgery patients	67,848 (2779)	OR 1.04 (0.25 to 4.34)
		Abdominal surgery patients	79,101 (2633)	OR 0.38 (0.22 to 0.65)
		Cardiovascular surgery patients	128,038 (6006)	OR 0.54 (0.40 to 0.73)
	D’Apuzzo *et al.* 2012 ^166^	Orthopedic surgery patients ^#^	359 (19)	OR 1.9 (1.3 to 2.8)

OR = odds ratio; 95% CI = 95% confidence interval; ICU = intensive care unit; AHI = apnea hypopnea index; LOS = length of hospital stay; # = not controlled for obesity; OSA = obstructive sleep apnea

*(Systematic review of 622 references published later than 2009 retrieved using MedLine search term “sleep apnea postoperative complications” – www.pubmed.org)*

Yet some studies suggest a decreased risk of postoperative mortality in patients with a known diagnosis of sleep apnea ^34, 35, 43^. Ischemic preconditioning was hypothesized to be involved in this protective effect of OSA, despite higher rates of cardiovascular comorbidities ^44, 45^. Ischemic preconditioning is an experimental strategy during which exposure to short, non-lethal episodes of ischemia results in attenuated tissue injury from ischemia and reperfusion ^46^. The underlying mechanisms may include increased blood vessel collaterality ^47^ and reduced oxidative stress ^48^. Recent studies found patients with OSA to have less severe cardiac injury after acute non-fatal myocardial infarction ^49^. Protective preconditioning from OSA may not be limited to the heart muscle, but may also have beneficial effects in the kidney and the brain ^50– 52^.

It is important to note that published studies investigating the effect of OSA on postoperative mortality are based on retrospective chart review. These retrospective analyses used diagnostic coding of OSA as an independent variable. These studies did not control for intraoperative predictors of postoperative complications, such as blood loss, anesthetics used, and mode of mechanical ventilation ^53, 54^. Therefore, one can assume that the true impact of OSA on postoperative outcomes remains unclear.

Additionally, one could argue that patients already diagnosed with OSA might receive more careful postoperative management. Longer time to extubation ^55, 56^ and increased utilization of non-invasive ventilation have been reported in OSA patients ^33^. Furthermore, it is a challenge to isolate the effect of OSA from the known adverse perioperative outcome of other comorbidities seen in patients with OSA, such as hypertension, diabetes, dyslipidemia, and obesity ^57– 59^. Although randomized controlled trials are important, such trials are unlikely to be feasible. Given that postoperative pulmonary complications are rare and multifactorial, and that the phenotypes of OSA differ by patient, it is difficult to undertake a trial that can capture all the nuances of this question. Observational studies reflect the real world practice of anesthesiologists and allow for the large sample size that is needed to be able to make inferences on the ideal settings for specific patient populations. Despite the limited data on OSA as an independent perioperative risk factor ^60^, it is intuitive to conclude that OSA patients are at risk of developing severe perioperative complications. Therefore, identification and optimal perioperative management of OSA patients is mandatory.

## Why does OSA occur and how can the perioperative setting affect OSA?

### Upper airway physiology and pathogenesis of obstructive sleep apnea

The respiratory pump consists of an anatomically diverse group of muscles including thoracic wall muscles, the diaphragm, and other muscles of the trunk ^61^. The contraction of these muscles increases the thoracic volume, and the lung generates negative intra-thoracic pressure. That negative pressure translates to a negative intraluminal UA pressure and thereby results in inspiratory airflow. If the negative UA pressure drops below a critical value (Pcrit), the UA collapses ^62, 63^. In contrast to healthy controls, Pcrit is positive (>0 cmH _2_O) in OSA patients when paralyzed ^64^ or sedated, and UA dilator muscle activity is required to maintain airway patency ^65^.

**Figure 1.**
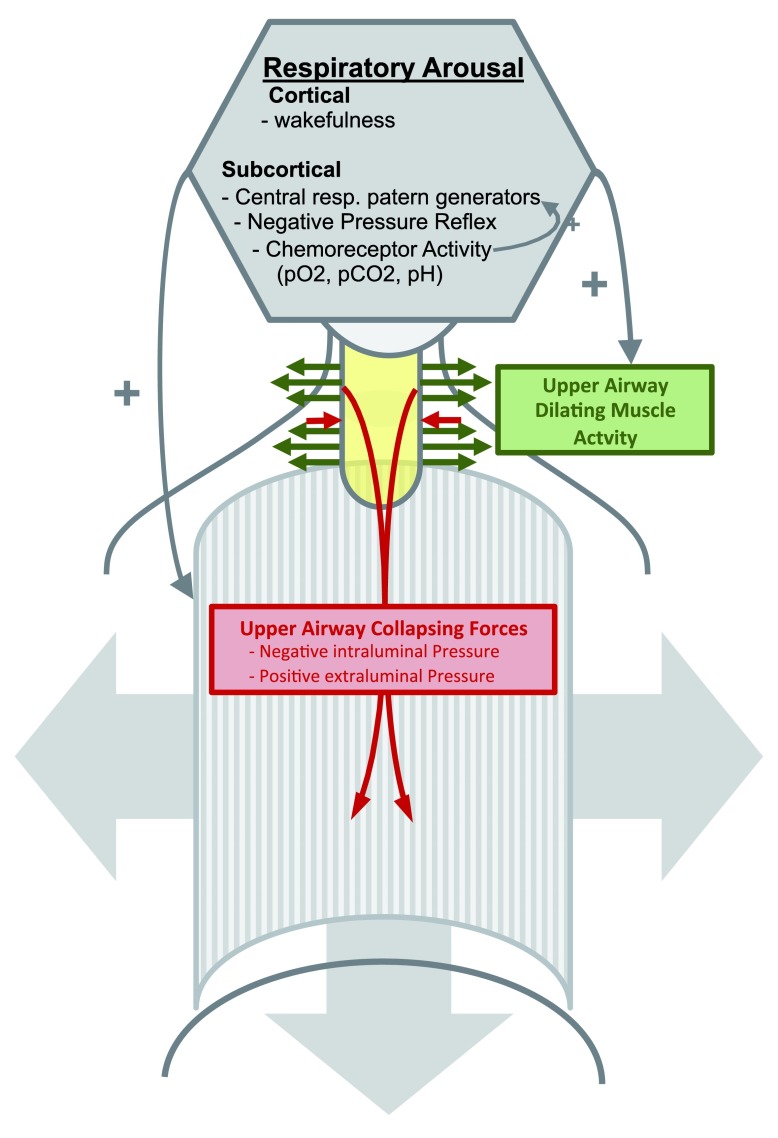
Perioperative upper airway patency. Respiratory arousal (grey hexagon) consisting of cortically and subcortically generated excitatory activity increases airway dilator muscle activity, thereby increasing upper airway dilating forces (green arrows). This counteracts the upper airway constricting forces (red arrows) generated by surrounding tissue pressure and negative intraluminal pressure during inspiration. (UA=upper airway yellow).

The activity of UA dilating muscles depends on neuronal innervation from subcortical and cortical brain regions that are modulated by physiological feedback and feed forward mechanisms. Subcortical regions of the brainstem and midbrain receive inputs for peripheral and central chemoreceptors that are sensitive to partial pressures of oxygen and carbon dioxide levels. Breathing and UA patency therefore respond to changes in gas exchange ^66^. Excitatory inputs from the UA, in response to a negative pressure, stimulate UA motor neurons to compensate for pressure changes across the respiratory cycle ^67, 68^. Respiratory pattern generators in the brainstem provide further excitatory input to the UA motor neurons and increase UA stability just prior to inspiration. While these subcortical regulatory circuits are effective, cortical inputs to the UA motor neurons are strongly connected to wakefulness ^69^. During wakefulness, serotonergic and noradrenergic neurons send excitatory inputs to the UA motor neurons ^70– 73^, resulting in increased UA dilator muscle activity. Yet, upon sleep onset, this neuronal input disappears and puts the UA at risk for collapse ^69, 75, 76^. UA collapse results in desaturations and an increase in the work of breathing that triggers cerebral arousal from sleep. The increased excitatory input to the UA motor neurons resulting from the sleep arousal reestablishes UA patency ^77^ ( Figure 1).

While the main source of airway collapsing forces is the negative intraluminal pressure during inspiration, additional predisposing factors increase the risk for airway collapse in OSA. UA anatomy ^78^ (e.g. hereditary reduction in the size of the retropalatal and retroglossal airway) ^79, 80^ and age ^81, 82^ are predisposing factors that cannot be altered by intervention. In contrast, increased extraluminal volume due to obesity (e.g. neck circumference) increases the risk of UA collapse ^81^ and can be improved by weight loss ^83^. Yet sleep apnea is not restricted to obese patients ^84^ and other factors such as reduced UA muscle activity (e.g. due to sedatives or alcohol) ^85^ may lead to decreased UA patency and apneas during sleep ^86, 87^.

### Perioperative factors affecting upper airway patency

During the perioperative period, multiple factors affect UA patency and increase the risk for UA obstruction, especially in patients with OSA ^88^. Mechanical loads to the collapsible segments of the retropalatal and retropharyngeal UA (e.g. postoperative hematoma ^89, 90^, peripharyngeal inflammation, and edema, e.g. due to fluid overload and rostral fluid shift ^91– 94^) lead to physical compression of the airway. Airway patency may also be affected by intubation and extubation. Supine positioning during surgery and the immediate postoperative period reduces lung volume and oxygen saturation. Reduced lung inflation due to pain-induced splinting and pharmacologic agents can limit tracheal traction on the UA and promote collapse ^94– 99^. Furthermore, decreased respiratory muscle function (i.e. diaphragm and intercostal muscles) results in impaired expansion of the lung and often occurs after surgery ^100^. Studies in the ICU have shown that systemic inflammation and mechanical ventilation can both dramatically reduce diaphragmatic function ^101, 102^.

Beyond these physical factors, pharmacological agents that are routinely applied during and early after anesthesia also affect breathing and UA patency in a dose-, muscle type- ^103^ and agent- ^104– 106^ dependent manner. Negative effects on breathing and UA patency were observed across three classes of gamma-aminobutyric acid (GABA)-ergic narcotics (volatile ^105, 107^, propofol ^104, 106, 108, 109^, and benzodiazepine ^110, 111^) and have impairing effects on UA dilator muscles. In contrast, ketamine ^112^ might have protective effects. In specific groups of patients (those with high loop gain), barbiturates may have protective effects on UA patency ^113, 114^. However, it is unclear if the latter is clinically meaningful in a perioperative scenario where decrease in ventilatory drive as a consequence of respiratory depressant drugs such as opioids may be more relevant in the pathophysiology of postoperative sleep apnea.

The underlying mechanisms of pharmacological agents on breathing are diverse ^106^. Dose-dependent increases in collapsibility of the UA through depressed respiratory drive, direct inhibition of UA dilator muscle activity (e.g. propofol) ^104^, and reduced responsiveness of UA dilator muscles to negative pressure (e.g. isoflurane) ^105^ have been shown for all GABAergic drugs, but N-methyl-D-aspartate (NMDA) antagonistic drugs such as ketamine and nitrous oxide may have respiratory protective effects, at least in the low-dose range. Nishino and colleagues investigated the differential effects of anesthetics and found greater dampening of hypoglossal nerve input relative to the phrenic nerve ^115^. Since this results in greater impairment of UA dilator muscles compared to respiratory pump muscles, the effects can lead to increased risk for UA collapse. In contrast, ketamine has been found to reduce neural input to the UA dilator muscles and equally to respiratory pump muscles. Ketamine’s effect on the UA dilator muscles was less when compared to GABAergic anesthetics ^115^. Studies in rats have demonstrated dissociation between loss of consciousness and UA dilator muscle function under ketamine anesthesia ^112^. Taken together, these studies suggest that patients with OSA, who have preoperative UA instability, may be at a heightened risk of UA collapse when under the influence of some, but not all, anesthetics. The unique effects associated with ketamine suggest that some anesthetic agents may be a safer choice for patients with OSA. However, prospective clinical studies in surgical patients with OSA are still required to confirm this preclinical finding and investigate the resulting effects on postoperative outcome.

In addition to reducing arousal and inducing loss of consciousness during surgery with medication, the anesthetist needs to apply neuromuscular blocking agents (NMBAs) carefully to cause muscle relaxation and optimize surgical conditions. The effects of NMBAs may outlast the duration of the surgical procedure. Postoperative residual neuromuscular blockade (rNMB) can affect postoperative respiratory outcome ^116, 117^ and has been reported to occur frequently after surgery ^118– 120^. rNMB as well as neostigmine reversal may also be associated with an even higher risk of complications in OSA patients. UA and respiratory pump muscles differ in their sensitivity to the effects of NMBAs ^121, 122^. These differences may lead to imbalanced activation of pump and dilator muscles, thereby generating excessive negative intraluminal pressure. Weakened UA dilator muscles would be unable to compensate for the excessive negative pressure. Even at levels that produce minimal blockade (as measured by train-of-four ratio 0.5 to 1), NMBAs increased UA collapsibility and impaired the compensatory genioglossus response to negative pharyngeal pressure challenges ^117^. Studies in surgical patients have demonstrated the dose-dependent association between intermediate-acting NMBAs and postoperative respiratory complications. That effect was shown to persist despite neostigmine-based reversal of neuromuscular blockade at the end of surgery ^42, 54, 123^. Although OSA patients should be more vulnerable to the effects of NMBAs and reversal agents ^117, 121, 124^, population-based studies do not currently exist.

Following surgery, opioids are commonly used for the management of postoperative pain. The use of opioids has been increasingly identified as a contributor to postoperative exacerbation of sleep-disordered breathing ^94, 125, 126^. OSA patients show a lower pain threshold ^127– 130^ and increased sensitivity to the respiratory depressant effects of opioids ^130^, both of which are of particular relevance in the postoperative setting. Increased UA resistance has been described in cats after opioid application ^131^ and may be mediated by direct inhibition of hypoglossal motor neurons with suppressed genioglossus activity ^132^. Therefore, the use of opioids during and immediately after surgery can be an important perioperative factor to consider in patients with OSA. Some data suggest that some interventions such as elevated upper body position ^94^ or continuous positive airway pressure (CPAP) ^133^ can ameliorate the respiratory depressant effects of opioids.

Finally, following surgery, patients commonly experience disrupted, reduced, and poor-quality sleep. Sleep fragmentation can reduce the rapid eye movement (REM) sleep stage ^134, 135^. Following sleep deprivation and fragmentation, a rebound effect with increased amount of REM sleep can be seen a few nights after surgery ^134, 136^. REM sleep is primetime for sleep apnea, since it is associated with muscle atonia and impaired respiratory arousal ^137^. Sleep deprivation may also contribute to the development of delirium, further disrupting sleep patterns and cortical arousals ^138^.

## How to manage patients with OSA perioperatively

Despite the high prevalence of OSA in surgical populations, standardized guidelines for the safe handling of this patient population are limited ^1^. The imperative is to provide the highest level of quality care while scheduling surgery in a timely manner and minding the expanding cost of providing care. Healthcare resources need to be optimally allocated to improve patients’ safety without undue economic impact. Given these restrictions, it is probably not feasible to conduct a sleep study on each patient scheduled for surgery, and there is so far no data indicating that a preoperative sleep medicine consultation improves patient safety. However, a stepwise approach for the detection of patients at risk of sleep apnea may help guide the need for diagnostics and treatment.

Patients should be tested for the risk of sleep apnea, and there are several validated scores available for preoperative testing such as the STOP BANG questionnaire ^139, 140^. We have recently developed an OSA screening instrument that is supposed to be applied to patients who have not been scheduled to see an anesthesiologist prior to the day of surgery. The Score for Preoperative prediction of OSA (SPOSA) can be used based on data available preoperatively in the electronic medical record without the need to take a physical exam ^141^. Once patients are identified to be at a high risk for sleep apnea in the perioperative setting, they demand special attention and care during the perioperative period and anesthesia. To some extent, the need for additional testing may depend on the perioperative risk of the scheduled surgical intervention. Data on the optimal intraoperative and perioperative management of sleep apnea is still limited, but OSA patients undergoing surgery and anesthesia with high risk of morbidity should receive specialized treatment during the perioperative period based on the best available local evidence and experience level.

**Figure 2.**
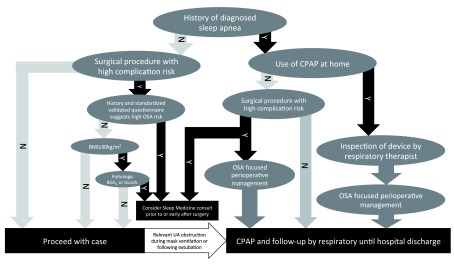
Algorithm for the perioperative detection and management of patients with sleep apnea (Y=yes; N=no). (modified from Zaremba S, Chamberlin NL, Eikermann M in Miller's Anesthesia 8th Edition, by Miller RD. Eriksson LI, Fleisher LA, Wiener-Kronish JP, Cohen NH, Young WL).

The use of a standardized algorithm, such as the one developed by physicians at the Massachusetts General Hospital in Boston ( Figure 2), may help identify and manage OSA patients facing surgery. In patients with known OSA who have been prescribed CPAP, the use of CPAP is continued during the postoperative period (e.g. in the recovery room). A respiratory therapist may visit the patient preoperatively or postoperatively in the recovery room to make sure the device and its interface function properly. During the postoperative period, when the patient is under lingering effects of anesthetics, CPAP treatment under close guidance of respiratory therapists reduces the number of respiratory events ^142^ and improves breathing early after surgery ^126^. Furthermore, CPAP may even improve postoperative hemodynamics (i.e. blood pressure variance) in patients who are not hypovolemic ^142^. CPAP treatment under close guidance of respiratory therapists likely reduces postoperative complications in OSA patients ^143^. However, successful perioperative CPAP treatment needs a close collaboration among patients, surgeons, anesthesiologists, and respiratory therapists, and sleep physicians may need to be consulted in selected patients.

For patients not previously diagnosed with OSA, clinical management should be performed based on risk stratification. Patients undergoing surgical procedures with moderate to high risk of perioperative complications should be stratified based on OSA risk. Risk for occult or undiagnosed OSA is based on the clinical assessment (e.g. symptoms and/or comorbidities of sleep apnea; see Table 3) and the use of standardized validated questionnaires (e.g. Berlin questionnaire ^144^, STOP questionnaire ^140^, P-SAP score ^139^, or the SPOSA ^141^). Note that these questionnaires have been validated for the identification of OSA, but not other forms of sleep-disordered breathing (e.g. obesity hypoventilation or central sleep apnea) ^145^. Additional testing, such as arterial blood sampling, is required to detect hypercapnia (increased blood carbon dioxide). However, blood gas analysis is not typically included in the standard preoperative workup for surgical patients and might not be available in some settings. In these cases, venous serum bicarbonate concentration, as available on most biochemistry profiles, might be a helpful screening tool for an occult, chronic, respiratory acidosis. A serum bicarbonate level greater than 27 mmol/l has been shown to be highly sensitive (92%) for an elevated arterial partial pressure of carbon dioxide. An elevated bicarbonate level accompanied by mild hypoxemia (peripheral oxygen saturation of 94%) may also indicate high risk of obesity-related alveolar hypoventilation ^145, 146^.

**Table 3.** Symptoms and comorbidities of sleep apnea.

*Symptoms:*
- Snoring (especially when loud and irregular) - Witnessed apneas - Frequent awakening (with/without choking sensation) - Nycturia - Nocturnal choking sensations - Morning headache - Daytime sleepiness - Daytime dysfunction
***Comorbidities:***
- Obesity - Arterial hypertension - Atrial fibrillation - Diabetes mellitus - Dyslipidemia

Patients deemed at high risk for OSA and/or obesity hypoventilation syndrome based on these screening tools may require sleep medicine consultation prior to or following anesthesia. The sleep specialist can help determine the role of a sleep study (home vs. laboratory based), start therapy with positive airway pressure therapy (e.g. auto-titrating CPAP), and develop strategies to “desensitize” patients to the mask and pressures prior to or following an elective surgical procedure.

Throughout the perioperative period, special attention should be paid to patients with confirmed OSA or high-risk patients under special circumstances. In these patients, specific methods should be used during intubation, intraoperatively, during and early after extubation, and during post-anesthesia care unit (PACU) treatment including fluid management, patient positioning, neuromuscular blockade, protective ventilation ^53, 147^, pain management, and choice of anesthesia type and anesthetic ( Table 4). However, the currently available data on the choice of anesthetic for the OSA patient facing surgery are limited, but the choice of a sedative agent to be applied intraoperatively can affect OSA patients and increase their risk for postoperative complication, as the majority of currently available anesthetics have been found to impair UA patency (see above). However, the supplementation of anesthesia with NMDA receptor antagonists might be a superior alternative during some surgical and interventional procedures in OSA patients. Ketamine can be particularly useful for pain control during short procedures such as dressing changes in burn patients, endoscopic procedures, and small surgical interventions. Compared to the majority of anesthetics, low-dose ketamine preserves airway reflexes and maintains respiratory drive. Ketamine might even stimulate breathing when applied in low to moderate doses in combination with other anesthetics. Data from animal studies and clinical studies in non-OSA patients showed an increase in respiratory rate ^112, 148, 149^, tidal volume ^112^, and minute ventilation ^112^ compared to other anesthetic agents, to sleep, and even to wakefulness ^149^. In clinical studies, combined anesthesia with ketamine was associated with improved oxygen saturation ^150^ and reduced time with supplemental oxygen ^151^ compared to regimes not including ketamine. In addition, ketamine’s positive effect on UA patency due to increased activity of UA dilating muscle activity ^112^ might be of special benefit in patients with OSA. Other beneficial effects making ketamine a favorable anesthetic agent for OSA patients include ketamine’s analgesic effects, as ketamine infusions have significantly reduced the requirements of opioids in patients with cancer and non-cancer pain ^152^. However, currently, no studies investigating the effect of ketamine on breathing in OSA patients have been reported. Randomized controlled trials are required to investigate if ketamine and other NMDA receptor antagonists have similar beneficial respiratory effects in patients with OSA and if these effects translate to improved postoperative outcome.

**Table 4.** Perioperative OSA Bundle (adopted from Dr Shiroh Isono, personal communication with Dr Matthias Eikermann).

***Preanesthesia Period*** • Consider regional anesthetic techniques that minimize the chance of postoperative sedation	
***Induction Strategy*** • Monitoring: capnogram, tidal volume measurement • Sniffing position • Reverse Trendelenburg position • Consider intubation without non-depolarizing neuromuscular blocking agent (NMBA), consider succinylcholine • Triple airway maneuver with two hands • Lung recruitment maneuvers immediately after intubation and apply positive end-expiratory pressure (PEEP) for maintaining lung volume during surgery
***Intraoperative Management*** • Protective ventilation with PEEP • Short-acting anesthetics and narcotics preferred • Avoid high-dose steroidal NMBA • Use neuromuscular transmission monitoring • Whenever possible, use of sedatives and narcotics should be reduced • Agents with reduced impairing effect on upper airway patency might be considered (e.g. ketamine, pentobarbital) • Neuromuscular blockade should be monitored and residual neuromuscular blockade should be reversed
***Extubation and Post-Anesthesia Care Unit*** • Patient should be able to cooperate before extubation. Consider positioning of patients in PACU • Bed: upper body should be elevated by 45 degrees, lateral position preferred to minimize gravitational effects on the upper airway • In case of impaired respiratory function, a plan needs to be defined and documented for monitoring and treatment, including the consideration of non-invasive ventilation • Patients will be discharged to an unmonitored environment or home when they meet discharge criteria: • Vital signs within 20% from baseline • Adequate treatment of nausea • Acceptable pain and aldrete score • Passed room air challenge test	
***Pain treatment*** • Consider non-steroidal anti-inflammatory drugs to reduce opioid use whenever possible, if not contraindicated • Use caution when combining opioids with sedatives or hypnotics	

Other considerations for patients with high risk of OSA include the application of neuromuscular blockade. Thus, high doses of NMBAs should be avoided intraoperatively, as these may facilitate rNMB and increase risk of postoperative respiratory complications ^42, 121, 153, 154^. If used, quantitative neuromuscular transmission monitoring is highly recommended to reduce the risk of residual neuromuscular blockade and detect neuromuscular blockade persisting beyond surgery. Reversal of rNMB by administration of a cholinesterase inhibitor (e.g. neostigmine) should be considered when rNMB is present, but the reversal agent has to be titrated carefully, since inappropriate (high-dose) neostigmine reversal has been shown to impair UA function in animals and humans ^122, 155^.

Patients undergoing surgical and anesthetic procedure with low risk of postoperative complications may receive standard postoperative care. However, in cases where UA obstruction occurs during intubation, extubation, or the early postoperative period ^88^, the postoperative application of CPAP with monitoring by a health care professional should be considered. Autotitrating CPAP (APAP) devices, which provide variable levels of pressure based on flow limitation, peak flow, vibration (snoring), and airway impedance ^156^, are a reasonable option for the CPAP-naïve patient ^157^. However, some studies indicate APAP without supervision by respiratory therapists does not improve oxygen saturation or outcome in all cases ^158, 159^, and difficulties with use can result in a low adherence to the therapy ^160^.

Following surgery, specialized attention should continue for patients diagnosed with, or at high risk for, OSA. If adherence to CPAP is limited in CPAP-naïve patients, the use of positional therapy may be a feasible option. Positional therapy, however, has been shown to be less effective compared to CPAP ^161^. Yet UA patency can easily be improved by elevation of the upper body, especially in the recovery room and during sleep ^94, 97^. Alternatively, a lateral body position can be used to minimize gravitational impact on UA whenever acceptable ^98^.

Opioids are commonly used for the control of surgical pain during and after anesthesia. This is of special importance in OSA patients who have been found to require higher doses of opioids for adequate pain control ^162^. While pain management may improve the use of the respiratory pump, these analgesics induce a dose-dependent impairment of UA dilator muscle activity ^94, 132, 163^. Recent data from our group and others indicate that treatment of OSA patients with CPAP early after surgery improves sleep apnea and mitigates negative effects of opioid application. An alternative, such as non-steroidal anti-inflammatory drugs (NSAIDs) or regional anesthesia with local anesthetics, should also be considered.

Given these perioperative factors, the transfer of an OSA patient from the recovery room or ICU to an unmonitored floor should be carefully considered. Patients should not be moved until their vital signs have recovered to values similar to pre-anesthesia baseline and after passing a room air challenge test. Furthermore, adequate treatment of nausea and pain should be accomplished, preferably by NSAIDs, prior to transfer.

## Conclusion

When caring for OSA patients facing surgery, the therapeutic team needs to be aware of the increased risk for post-anesthesia respiratory complications. While these complications are not associated with increased mortality risk, the morbidity of preventable complications may lead to undesired expenses, jeopardize available resources, and may lead to an increased hospital readmission rate. Since the currently available literature on perioperative management of OSA patients is still limited, additional clinical and basic research in this area is needed to improve the safety of OSA patients undergoing anesthesia.

We hypothesize that early recognition and treatment of sleep apnea reduces perioperative complications. Further research is needed to confirm this clinical suspicion and support the use of diagnostic or therapeutic algorithms for these patients.

Pending that research, an institution-specific plan (based on setting, personnel, equipment, medications, and resources) needs to be established for the identification, testing, monitoring, and care of the surgical population. The plan should include (1) stepwise preoperative screening procedures for OSA, (2) an optimized anesthesia regimen and sedation protocol for this high-risk group which eliminates drug-induced respiratory depressant effects at the end of the case, (3) intraoperative neuromuscular monitoring with goal-directed reversal of rNMB, (4) a protocol for the use of CPAP therapy in the recovery room, (5) optimal opioid titration for postoperative pain control, and (6) specific discharge criteria for transfer to the unmonitored ward.

## Abbreviations

CPAP           -        continuous positive airway pressure

GABA          -        gamma-aminobutyric acid

NMBA         -        neuromuscular blocking agents

NMDA         -        N-methyl-D-aspartate

NSAID         -        non-steroidal anti-inflammatory drugs

OSA             -        obstructive sleep apnea

REM sleep   -         rapid eye movement sleep

rNMB          -         residual neuromuscular blockade

UA               -         upper airway
